# Infection with carcinogenic helminth parasites and its production of metabolites induces the formation of DNA-adducts

**DOI:** 10.1186/s13027-019-0257-2

**Published:** 2019-11-29

**Authors:** Maria João Gouveia, Paul J. Brindley, Gabriel Rinaldi, Fátima Gärtner, José M. C. da Costa, Nuno Vale

**Affiliations:** 1 Center for the Study of Animal Science, CECA-ICETA, University of Porto, Praça Gomes Teixeira, Apartado 55142, 4051-401 Porto, Portugal; 2 Department of Molecular Pathology and Immunology, Institute of Biomedical Sciences Abel Salazar (ICBAS), University of Porto, Rua de Jorge Viterbo Ferreira 228, 4050-313 Porto, Portugal; 3 i3S, Instituto de Investigação e Inovação em Saúde, University of Porto, Rua Alfredo Allen, 208, 4200-135 Porto, Portugal; 4 Department of Microbiology, Immunology and Tropical Medicine and Research Center for Neglected Diseases of Poverty, School of Medicine and Health Sciences, George Washington University DC, Washington DC, 20037 USA; 5 Wellcome Sanger Institute, Wellcome Genome Campus, Hinxton, Cambridge, CB10 1SA UK; 6 Institute of Molecular Pathology and Immunology of the University of Porto (IPATIMUP), Rua Julio Amaral de Carvalho, 45, 4200-135 Porto, Portugal; 7 National Health Institute Dr. Ricardo Jorge (INSA), Rua Alexandre Herculano, 321, 4000-055 Porto, Portugal; 8 Laboratory of Pharmacology, Department of Drug Sciences, Faculty of Pharmacy, University of Porto, Rua de Jorge Viterbo Ferreira, 228, 4050-313 Porto, Portugal

**Keywords:** (max. 10): Helminth infection, Malignancy, Schistosomiasis, Opisthorchiasis, Cytochrome P450, Oxysterol

## Abstract

**Background:**

Infections classified as group 1 biological carcinogens include the helminthiases caused by *Schistosoma haematobium* and *Opisthorchis viverrini*. The molecular mediators underlying the infection with these parasites and cancer remain unclear. Although carcinogenesis is a multistep process, we have postulated that these parasites release metabolites including oxysterols and estrogen-like metabolites that interact with host cell DNA*.* How and why the parasite produce/excrete these metabolites remain unclear. A gene encoding a CYP enzyme was identified in schistosomes and opisthorchiids. Therefore, it is reasonable hypothesized that CYP 450 might play a role in generation of pro-inflammatory and potentially carcinogenic compounds produced by helminth parasites such as oxysterols and catechol estrogens. Here, we performed enzymatic assays using several isoforms of CYP 450 as CYP1A1, 2E1 and 3A4 which are involved in the metabolism of chemical carcinogens that have been associated with several cancer. The main aim was the analysis of the role of these enzymes in production of helminth-associated metabolites and DNA-adducts.

**Method:**

The effect of cytochrome P450 enzymes CYP 1A1, 2E1 and 3A4 during the interaction between DNA, glycocholic acid and taurochenodeoxycholate sodium on the formation of DNA-adducts and metabolites associated with urogenital schistosomiasis (UGS) and opisthorchiasis was investigated *in vitro*. Liquid chromatography/mass spectrometry was used to detect and identify metabolites.

**Main findings:**

Through the enzymatic assays we provide a deeper understanding of how metabolites derived from helminths are formed and the influence of CYP 450. The assays using compounds similar to those previously observed in helminths as glycocholic acid and taurochenodeoxycholate sodium, allowed the detection of metabolites in their oxidized form and their with DNA. Remarkably, these metabolites were previously associated with schistosomiaisis and opisthorchiasis. Thus, in the future, it may be possible to synthesize this type of metabolites through this methodology and use them in cell lines to clarify the carcinogenesis process associated with these diseases.

**Principal conclusions:**

Metabolites similar to those detected in helminths are able to interact with DNA in vitro leading to the formation of DNA adducts. These evidences supported the previous postulate that imply helminth-like metabolites as initiators of helminthiases-associated carcinogenesis. Nonetheless, studies including these kinds of metabolites and cell lines in order to evaluate its potential carcinogenic are required.

## Background

More than 20% of cancers in the developing world are caused by infections, including helminthiases [1]. In addition to the direct impact on the development, health, and prosperity of populations in endemic regions, chronic infection with the liver fluke *Opisthorchis viverrini* and the blood fluke *Schistosoma haematobium* leads to cholangiocarcinoma (CCA), bile duct cancer, and squamous cell carcinoma (SCC) of the urinary bladder, respectively. The chronic infection with these helminths is recognized by International Agency for Research on Cancer (IARC) as a definitive cause of cancer [2].

These helminths produce and excrete metabolites, including estrogens and oxysterols, that appear capable of oxidation of host DNA, in turn leading to the formation of depurinating DNA adducts and mutations in the genome of adjacent tissues acting as initiators of carcinogenesis [3–7]. The high-performance liquid chromatography coupled with mass spectrometry (LC-MS/MS) analysis of urine from individuals with urogenital schistosomiasis (UGS) and bladder cancer revealed the presence of specific metabolites which may represent biomarkers for diagnosis and prognosis of SCC [6]. These metabolites were also identified in developmental stages of *S. haematobium* and *Opisthorchis viverrini* [5–8]. Chronic infection with the liver fluke, *Opisthorchis felineus* may mimic the malignant transformation induced by *O. viverrini* infection producing metabolites that interact with host DNA. In addition, several DNA adducts were detected in biofluids from hamsters experimentally-infected with *O. felineus*. These findings may support the inclusion of the infection with the liver fluke *O. felineus* as group 1 carcinogens by the IARC [5].

The CYP450 enzymes are involved in several biological processes such as biosynthesis of estrogen, conversion of cholesterol into bile acids and its biotransformation [9, 10]. These enzymes include the CYP450 family members CYP 1A1, 2E1 and 3A4, involved in the metabolism of chemical carcinogens, associated with breast and endometrium cancer in humans [11]. A gene encoding a CYP enzyme was identified in schistosomes and opisthorchiids [12–14]. Accordingly, this family of enzymes might play a role in generation of pro-inflammatory and potentially carcinogenic compounds produced by helminth parasites such as oxysterols and catechol estrogens. The role of CYP 450 enzymes in the physiology and biochemistry of these flukes is less well understood. However, it might contribute to cell survival, drug resistance, maintenance and evolution of the host-parasite relationships [13]. In case of *O. felineus* the function of CYP450 has been linked to the excretory system of the parasite and possibly to metabolism and detoxification as biotransformation of endogenous substrates [13]. Nevertheless, it remains unclear whether trematode CYP enzymes catalyze the synthesis of proinflammatory and potentially carcinogenic compounds, e.g. oxysterols-like and catechol-like estrogen quinone-like metabolites known to be released by these flukes [6–8].

Previously, we have hypothesized that helminth-derived metabolites may be able to interact with host DNA [3, 4]. Here we conducted in vitro assays in order to evaluate the role of CYP450 in the formation of carcinogenic metabolites [5–8]. Due to difficulties in isolating the metabolites detected on developmental stages of the parasites or biofluids of infected individuals, we decided to use commercially-available compounds, i.e. glycocholic acid and taurochenodeoxycholate sodium. Thus, we exposed exogenous DNA to glycocholic acid and taurochenodeoxycholate sodium in the presence or absence of CYP 450. Subsequently, we identified the reaction products by liquid chromatography-mass spectrometry (LC-MS/MS).

## Materials and methods

### Chemicals

Acetonitrile (ACN) and formic acid (HF), HPLC grade, were obtained from Merck (Darmstadt, Germany). Glycocholic acid hydrated (G2878-100MG), calf thymus DNA (D1501-100MG), nicotinamide adenine dinucleotide phosphate (NADPH, N7506-25MG), dimethyl sulfoxide (DMSO, D-5879) and CYPExpress™ 1A1 (MTOXCE1A1-250MG), 2E1 (MTOXCE2E1-250MG) and 3A4 (MTOXCE3A4-250MG) were purchased from Sigma/Merck (Sintra, Portugal). Taurochenodeoxycholate sodium (20275) was purchased from Cayman Chemical (Ann Arbor, Michigan, USA).

### CYP450 activity, interaction of precursor metabolites with DNA and formation of DNA adducts

**Fig. 1:**
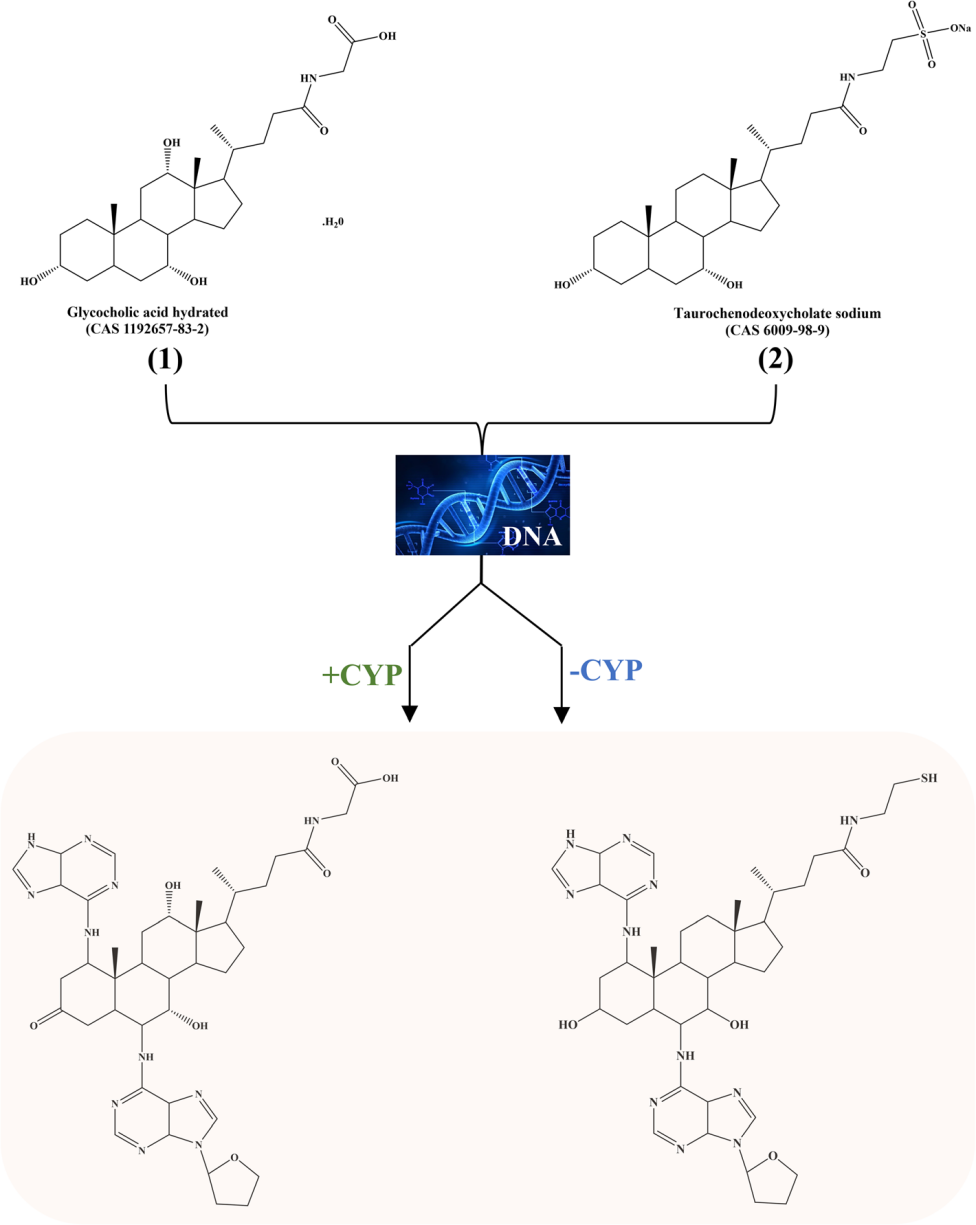
DNA adducts that we have implicated previously in schistosomiasis and opisthorchiasis [5–8]. Molecules (**1**) and (**2**) are presented as precursors. The ability of these compounds in interact with DNA in vitro leading to the formation of DNA adducts was investigated, along with a role for cytochrome P450 enzymes in formation of the adducts

The structures of compounds glycocholic acid (**1**) and taurochenodeoxycholate sodium (**2**) are shown in Fig. 1. Stock solutions of both compounds were prepared in DMSO. The compounds at final concentration of 100 μM were incubated with CYP1A1, CYP2E1 and CYP3A4 (0.2 μM) in the presence of 1.4 μM of NADPH and calf thymus DNA (3 mM) in 67 mM Na-K phosphate buffer (pH 7.2), in a total volume of 200 μL at 37 °C for 72 h [15, 16]. A control reaction was prepared as described above but without CYP450 enzymes. In addition, a well containing the matrix of reactions was also prepared with NADPH, calf thymus and Na-K buffer. Aliquots of the reaction mixture were collected at 24 and 72 h after the start of the reaction. The reaction was stopped by addition of two volumes of ethanol (EtOH) to precipitate DNA, which was recovered by centrifugation. Thereafter, the supernatant was subjected to analysis by LC-MS/MS. It should be noted that data originated from matrix sample were subtracted (in LC/MS) to eliminate its influence.

### Detection of metabolites and DNA-adducts by liquid chromatography mass spectrometry (LC-MS/MS) analysis

Detection and identification of metabolites and related DNA-adducts by LC-MS/MS was undertaken using an LTQ Orbitrap XL mass spectrometer (Thermo Fischer Scientific, Bremen, Germany), fitted with an ultraviolet (UV) photo diode assay (PDA) detector. Analysis of aliquots involved a single injection of 20 μL with an ACE Equivalence 5 C_18_ (75 mm Х 3 mm i.d.) column. The mobile phase consisted of 1% HF in water (A)/ACN (B) mixtures. Elution proceeded at a flow rate of 0.5 ml/min. Eluates were monitored for 10 min, run with a mobile phase gradient started with 80% A and 20% B. At that point, B was increased linearly to 55% over 5 min. The gradient was returned to the starting proportion at 9 min and equilibrated for one minute. Data were collected in positive electrospray ionization (ESI). The capillary voltage of the ESI was 28 kV, and its temperature was 310 °C, flow rates of the sheath gas and auxiliary gas (N_2_) were set to 40 and 10 (arbitrary units as provided by software settings), respectively, and gas temperature was 275 °C.

## Results

### Formation of glycocholic acid and taurochenodeoxycholate sodium metabolites and DNA adducts is independent of CYP 450

The chromatograms and ratio mass/charge (m/z) obtained by LC-MS/MS analysis are shown in Additional files 1 and 2. With regard to the starting compounds, the precursor glycocholic acid (**1**) with molecular mass [M + H] 466.32 was identified in all aliquots analyzed (Additional file 3). Additionally, product spectra as ratio mass/charge (m/z) 338 [M-3H_2_0-C_2_H_4_NO_2_ + H]^+^ and 412 [M-3H_2_0 + H]^+^ were identified as its metabolites, as previously described [17]. In contrast, taurochenodeoxycholate sodium (**2**) (m/z 522.28) was not detected.

Through the analysis of the data obtained for aliquots (Additional file 1), it was possible to observe that sample (+CYP) at 24 h and control (−CYP) at 72 h included more metabolites than (+CYP) at 72 h and (−CYP) at 24 h. Apparently, the number of metabolites on samples decreased through the course of reaction. The opposite effect was observed in the controls, i.e. at 24 h there were fewer compounds than at 72 h; 43 vs 77. For the samples (+CYP) during the course of the reaction, the number of compounds decreased, suggesting that the reaction in the presence of CYP450 was faster but might lead to some transformation and, in turn, result in fewer products. By contrast, in controls (−CYP) the number of compounds slightly increased during the reaction which suggested that reaction in the absence of CYP450 might be slower, and lead to the formation of an elevated number of compounds. In turn, this might indicate that the chemical transformation was less evident in controls (−CYP) (Additional file 1).

Nevertheless, some of the metabolites detected were common to 1) all aliquots; 2) samples (+CYP) (e.g. samples at 24 and 72 h); 3) control (−CYP) (e.g. control at 24 and 72 h); 4) samples or control at same time of reaction (e.g. sample and control at 24 h); 5) samples (+CYP) or control (−CYP) on different time of reaction (e.g. sample at 24 and control at 72 h); 6) to three different aliquots (e.g. sample at 24, controls at 24 and 72 h) (Additional file 2). Generally, the generated compounds were mostly metabolites of precursors in oxidized or catechol form and DNA-adducts (Additional files 2 and 3). Indeed, samples and controls at 24 h included several common metabolites that correspond not only to oxidized metabolites (e.g. m/z 628.30, Additional file 3) but also DNA-adducts (e.g. 718.41, Additional file 3). The highest number of DNA adducts was observed in the sample (+CYP) at 24 h; however, some of these DNA-adducts were also detected on control (−CYP) at 24 h. Similarly, some compounds either in oxidized form or as DNA-adducts detected in samples (+CYP) at 24 h were also present in controls (−CYP) at 72 h (e.g., m/z 734.40 and 801.43 Additional file 3). Since metabolites and related DNA-adducts were detected either in samples (+CYP) or controls (−CYP) at the same or at a different time of reaction, we hypothesize that their formation was independent of the presence of CYP450. Notably, the compounds that were common to three samples, (+CYP) at 24 and (−CYP) at 24 and 72 h were also DNA adducts (e.g., m/z 566.34). Curiously, in comparison (+CYP) at 24 and 72 h and (−CYP) at 72 h and (+CYP) 24 h and 72 h, most of the common compounds were fragments of precursors (e.g., m/z 403.27, Additional file 3).

**Fig. 2:**
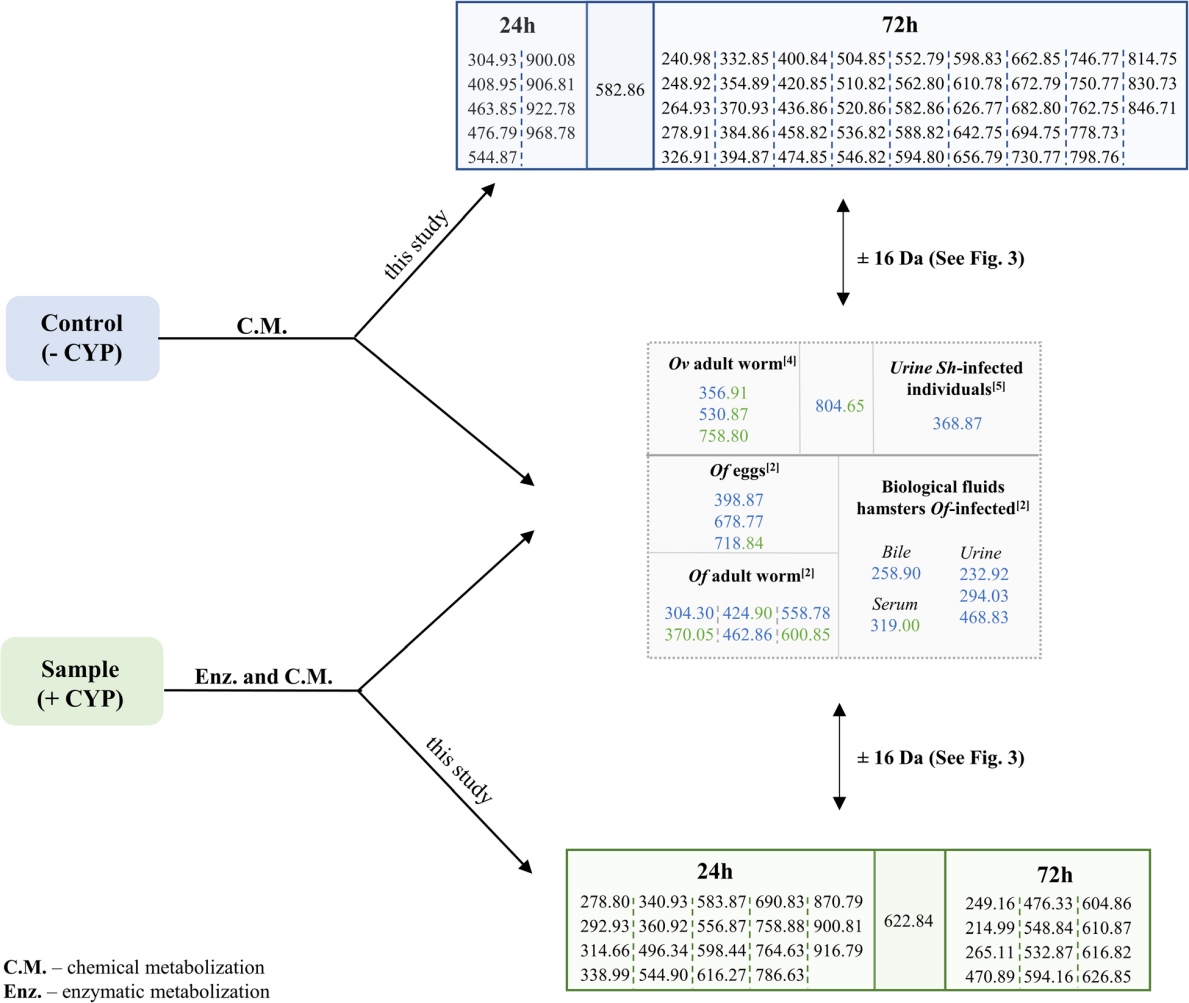
Exclusive m/z detected in control (−CYP) and samples (+CYP) during the course of the reaction. Several compounds that had been detected previously within developmental stages of parasites and biofluids from mammalian hosts were synthesized in vitro in this study

Comparing all exclusive metabolites obtained for all aliquots, we observed that controls (−CYP) 72 h had the most elevated number (56) but also the lowest at 24 h (10) (Fig. 2). It was expected that the number of compounds would be higher in samples (+CYP) than in the controls (−CYP). However, as noted above, this could suggest that the formation of metabolites and DNA adducts was independent of CYP450, and that most of the metabolites form through a chemical metabolization that occurs in the reaction mixture, and not by enzymatic catalysis (Fig. 2).

Generally, the exclusive compounds were mainly metabolites of precursors of glycocholic acid and taurochenodeoxycholate sodium, and most of them were in oxidized or catechol form [(e.g. m/z 541.21 at S24 h (+CYP); m/z 509.24 at C72 h (−CYP)]. Interestingly, at C24 h (−CYP) we did not observe exclusive metabolites in oxidized or catechol forms (Additional file 4). All aliquots except C72 h (−CYP) presented DNA adducts [e.g. m/z 616.34 at S24 h (+CYP); m/z 899.43 at C24h (−CYP); m/z 598.75 at S72 h (−CYP)]. Regarding the common metabolites, aliquots S24 h (+CYP) and C24 h (−CYP) shared not only metabolites in oxidized form as well as DNA-adducts (e.g. m/z 628). Similarly, this was observed on S24 h (+CYP) and C72h (−CYP) (e.g. m/z 743.40), and S72 h (+CYP) and C72 h (−CYP) (e.g. m/z 612.44) (Fig. 2; Additional file 4). Additional files 3 and 4 present the common and exclusive postulated structures of the metabolites and cognate DNA-adducts.

### The metabolites and DNA-adducts are similar to those identified during schistosomiasis and opisthorchiasis

**Fig. 3:**
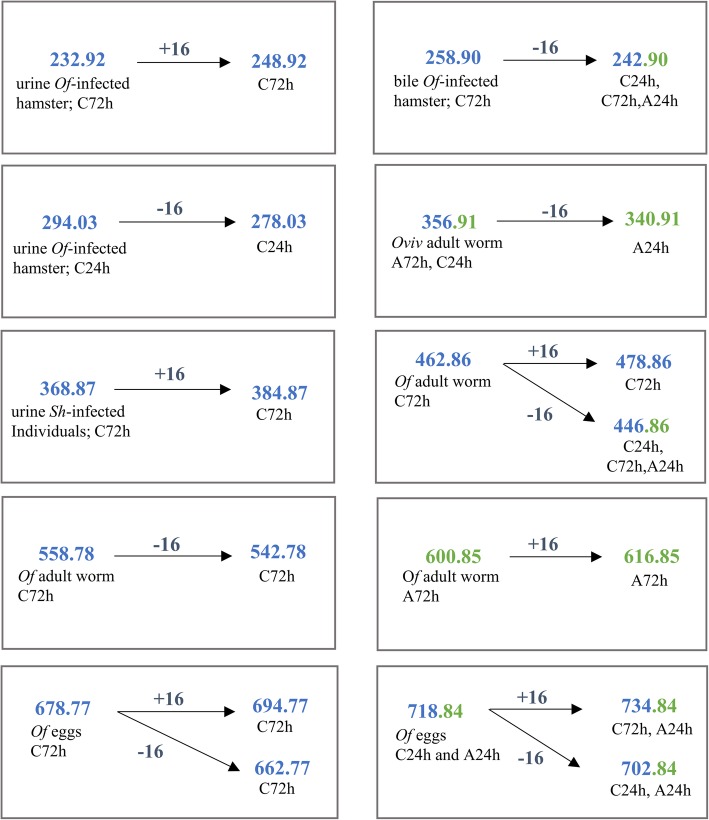
Mass/ratio (m/z) of metabolites and DNA adducts synthesized in vitro, and previously related to opisthorchiasis and schistosomiasis which undergo hydroxylation (± 16 Da)

In order to test if the in vitro assay leads to the synthesis of metabolites related to schistosomiasis [6] and opisthorchiasis [5, 7], we compared the current data with published findings [5–8]. The identities of common compounds were confirmed by comparison of their mass spectra and molecular mass (Fig. 2). Indeed, some metabolites and DNA-adducts synthesized during the present assays (e.g. m/z 600, 718, 804, Figs. 2 and 3) had been previously associated with these helminth infections [5, 7]. In addition, some metabolites detected here were identified as oxidized forms [M + 16] of those previously described for helminthic infection [5, 6, 7] (Figs. 2 and 3). Interestingly, most of these similar compounds were not DNA-adducts, but were precursor metabolites.

Additionally, most of these compounds were detected on control (−CYP) or were common to control (−CYP) and samples (+CYP) suggesting that CYP450 was not critical to produce electrophilic metabolites observed on flukes and infected individuals (Figs. 2, 3). Based on these findings, we could hypothesize that infection with the liver fluke might have a potential role in the formation of DNA-adducts.

In addition, during this in vitro study, it was possible to synthesize a new family of metabolites of glycocholic acid (**1**) and taurochenodeoxycholate sodium (**2**), as well as to demonstrate that these metabolites are able to interact with DNA forming DNA-adducts. It should be noted that some the metabolites and DNA adducts synthesized here correspond to those previously related to schistosomiasis and opisthorchiasis. Thus, we consider that data presented here reinforce the notion that this kind of compounds and the helminth-derived metabolites, are capable to interact with host DNA and lead to the formation of DNA-adduct. Therefore, these evidences support, at least in part our postulated [3, 4].

## Discussion

In this study, we performed an in vitro assay to investigate the generation of some of the metabolites and related DNA-adducts previously identified in the context of opisthorchiasis, schistosomiasis and their infection-associated cancers [5, 6, 8]. The precursors, glycocholic acid (**1**) and taurochenodeoxycholate sodium (**2**), were chosen based on previous data that demonstrated cognate DNA-adducts in developmental stages of parasites and in biofluids of human cases and/or rodent models of these helminth infections [5–8]. Using in vitro assays, we confirmed the ability of these compounds to interact with DNA leading to the formation of DNA adducts.

Bile acids exhibit a diverse spectrum of biological activity, being able to stimulate proliferation and tumor invasiveness, inhibit apoptosis and modify the promoter functions of genes involved in DNA synthesis, repair, and oxidative stress [18, 19]. Bile acids are associated to several carcinogen models, e.g. glycocholic acid was high in urine of people with hepatocellular carcinoma [20]. Additionally, in human esophageal cell lines, acid and bile salts, including glycocholic acid (**1**), caused elevated DNA damage [20]. CYP450 enzymes are involved in the generation of most chemical carcinogens that induce genotoxicity and DNA damage [10, 21, 22]. CYP450 play a critical role both in estrogen formation and its subsequent metabolism, namely, CYP1A1 and CYP3A which catalyze the hydroxylation preferentially at the 2-position, whereas CYP1B1 catalyzes the hydroxylation almost exclusive at the 4-position [23], leading formation of catechol estrogen quinone form that can react with DNA to form predominantly depurinating adducts.

We have previously hypothesized a similar pathway for the genesis of parasite-derived metabolites [4]. Here, we investigated the role of these enzymes in activation and/or oxidation of parent compounds. Although CYP450 enzymes might be involved in formation of some metabolites detected in samples (+CYP), apparently, they were not crucial during the metabolization of these compounds to form DNA-adducts, since they were detected in control aliquots (−CYP). The metabolites and oxidized forms detected here were probably generated by non-enzymatic autoxidation process [24, 25]. Nonetheless, some of oxidized metabolite were observed in samples (+CYP). Therefore, we cannot rule out that these enzymes play a role in the formation of oxidized metabolites since involvement of CYP450 in the oxidation of several compounds have been reported in previous study [26]. Apparently, previous activation is not necessary for interaction with DNA. Notably, the metabolites and DNA adducts that were previously described [5–8], and synthesized here for use in our in vitro assay, were generated in absence of P450. This suggests that the formation of these metabolites also might be independent of the parasite CYP450.

Carcinogenesis is a complex process in which normal cell growth is modified due interaction of multiple factors [27]. Malignant transformation follows a sequence of steps that include a pathogenic *stimulus* (biological and/or chemical) followed by a chronic inflammation, in turn provoking fibrosis and changes in the cellular microenvironment arising in a pre-cancerous niche [28]. In the cases of schistosomiasis and opisthorchiasis, we have postulated that these pathogens provide biological and chemical *stimuli* through parasite-derived metabolites, e.g. oxysterols and catechol estrogens, that can interact with host DNA to trigger a cascade of events ultimately leading to cancer [3–8]. Cholesterol, oxysterols and estrogens are closely related steroids [29]. These trematode parasites likely produce these steroid-like compounds for endogenous physiological and reproductive processes, but they| may also have evolved in concert with the host-parasite relationship. Oxysterols and/or catechol estrogens of trematode origin and/or precursors modified as the consequence of opisthorchiasis or UGS [2–8, 30] appear capable of modulating host metabolic pathways of steroid hormones and bile acids [31], and are potential initiators of carcinogenesis given that these metabolites are genotoxic in other systems [32]. Since these infection-related initiators of cancer and/or promoters might need to be metabolized for activation, their carcinogenicity may be specific for organ systems, tissues, and/or epithelia [1, 31].

Currently, little evidence supports the formation of DNA damage directly by bile acids. The information is controversial. Some authors state the impossibility of formation bile acid-related DNA adduct in vitro [33, 34] whereas, by contrast, others report that bile acids or conjugates of glycocholic acid (**1**) and taurochenodeoxycholate sodium (**2**) are responsible for the elevation of DNA damage on esophageal cell lines [20, 35]. Moreover, bile acids and the oxidized forms, oxysterols, are linked to development and progression of cancers of the pancreas, colon, lung and breast [3, 36, 37]. With respect to helminthic infections, opisthorchiasis is associated with an elevation of bile acids, including deoxycholic acid, which is a potent promoter in cholangiocarcinogenesis [7]. Indeed, several metabolites and DNA adducts detected in this study have been implicated during schistosomiasis and opisthorchiasis [5–8].

Helminth infections caused by *Opisthorchis* species and *S. haematobium* are directly linked to malignancy [2]. We have shown that metabolites excreted by parasites might act as chemical carcinogens and act as initiators of carcinogenesis, through interaction with host DNA [3, 4, 38]. Here, we demonstrated the in vitro generation of several metabolites of glycocholic acid (**1**) and taurochenodeoxycholate sodium (**2**), precursors of DNA-adducts related to opisthorchiasis and urogenital schistosomiasis [5, 6, 8]. Also, we confirmed the ability of these metabolites to interact with DNA leading to the formation of DNA-adducts. The activation and subsequent formation of DNA-adducts seem to be performed through a CYP450-independent pathway, at least CYP1A1, CYP1B1, and CYP3A4. Some of the metabolites previously detected in the helminths themselves and infected people, and in a rodent model of infection, were synthesized in this study (Figs. 2, 3). Future studies using informative cell lines can be expected to define the carcinogenic roles of these metabolites.

## Supplementary information

**Additional file 1.** Chromatograms obtained by LC-MS/MS of different aliquots analyzed.

**Additional file 2.** All m/z detected during analysis of LC-MS/MS of aliquots of samples and control during the course of reaction. Common and exclusive m/z detected for each of aliquots.

**Additional file 3.** Postulated structures for common compounds to different aliquots.

**Additional file 4.** Postulated structures for exclusive m/z detected on samples and control during course of reaction.

## Data Availability

All data generated or analyzed during this study are included in this published article.

## Abbreviations

*+CYP*: sample
*ACN*: acetonitrile
*CCA*: cholangiocarcinoma
*-CYP*: control
CYP450: *cytochrome P450*
*DMSO*: dimethylsulfoxide
*DNA*: deoxyribonucleic acid
*ESI*: electrospray ionization
*EtOH*: ethanol
*HF*: formic acid
*HPLC-MS/MS*: high performance liquid chromatographic mass spectrometer; *m/z*, ratio mass/charge
*IARC*: International Agency for Research on Cancer
*NADPH*: nicotinamide adenine dinucleotide phosphate
*PDA*: photo diode assay
*SCC*: squamous cell carcinoma
*UGS*: urogenital schistosomiasis
*UV*: ultraviolet
