# Supplementary material for: Infection with carcinogenic helminth parasites and its production of metabolites induces the formation of DNA-adducts
Source: Infect Agent Cancer. 2019 Nov 29;14:41. doi: 10.1186/s13027-019-0257-2 (PMC6884881; doi:10.1186/s13027-019-0257-2)
Supplement: Supplementary file 1 — Additional file 1. Chromatograms obtained by LC-MS/MS of different aliquots analyzed. [file 13027_2019_257_MOESM1_ESM.pdf]

All m/z detected

	(+ CYP)	(- CYP)
	**S24h**	**C24h**
m/z	240.99; 225.01; 319.00; 292.93; 370.05; 230.96; 360.92; 338.99; 314.66; 226.95 (242.93; 230.96; 220.94); 288.82 (294.94; 298.94; 310.91); 362.93 (356.91; 378.90; 361.13; 377.10; 372.98; 340.93); 430.91 (424.90; 446.89; 440.87; 498.90; 492.89; 514.88; 508.86; 486.87; 530.85); 566.89 (560.87; 583.87; 554.86; 576.85; 538.88; 544.90;598.44; 556.87; 606.87); 634.88 (628.86; 650.85; 644.83; 666.82; 690.83; 622.84); 696.85 (702.87; 718.84; 712.84; 734.81; 758.8); 764.63 (786.83; 770.86; 780.91; 758.82); 786.63 (780.81; 802.80; 838.84; 848.80); 832.82 (854.81; 870.79; 900.81; 916.79); 278.80; 256.90; 318.94; 390.94; 466.32 (77)	204.00; 225.01; 240.99; 226.96 (242.92; 230.97; 220.94); 288.92 (294.03; 310.93; 356.92; 298.95; 304.93; 272.99); 362.98 (378.92; 377.09; 372.90); 424.96 (430.91; 446.89; 440.88; 418.95; 408.95); 498.89 (492.88; 514.87; 508.86; 486.87; 475.20; 476.79; 463.85); 566.88 (560.87; 582.86; 576.86; 554.86; 544.87); 628.86 (634.87; 644.83; 650.85; 666.82); 696.85 (702.85; 718.83; 764.79; 770.83; 786.81; 780.91); 832.81 (838.82; 854.80; 848.79); 922.78 (968.78; 906.81; 900.08); 390.94; 606.85; 319.00; 466.32
	**S72h**	**C72h**
m/z	204.00; 225.47; 319.00; 230.96 (226.96; 220.93; 249.16; 214.99); 298.95 (288.92; 272.94; 265.11); 361.13 (377.10; 356.91; 402.90); 418.88 (424.90; 412.93); 475.32 (486.87; 476.33; 470.89; 492.88); 548.84 (542.89; 554.86; 532.87); 616.82 (622.84; 626.85; 600.85; 594.16; 604.86; 610.87; 804.65); 319.00 (361.13; 377.10; 356.10; 418.88); 466.32; 404.21	240.99; 204.00; 225.01; 226.95 (242.93; 248.92; 258.90; 232.92; 240.98; 264.93); 316.68 (310.92; 300.90; 294.92; 326.91; 278.91; 332.85); 378.90 (384.86; 368.87; 394.87; 362.92; 400.84; 361.10; 377.09); 446.88 (452.85; 436.86; 462.86; 468.83; 430.90; 458.82; 420.85; 478.84); 520.86 (514.87; 530.85; 536.82; 542.79; 504.85; 562.80; 498.89; 558.78; 552.79; 546.82; 510.82; 566.88); 610.78 (594.80; 598.83; 582.86; 626.77; 588.82; 650.85); 678.77 (694.75; 662.85; 666.82; 642.75; 682.80; 656.79, 672.79); 762.75; (746.77; 734.84; 730.77; 778.73; 798.76; 750.77); 830.73 (802.81; 804.79; 814.75; 846.71); 319.00; 466.32; 304.30; 404.21 (68)

Common:

- to all aliquots
- S24, C24h, C72h;
- S24h, S72h, C24h;
- S24h, C24h;
- S24, C72h
- S72h, C72h
- S24h, S72h
- S72h, C24h
- C24h, C72h
- S72h, C72h

S- sample
C- control

Exclusive

	(+ CYP)	(- CYP)
	**S24h**	**C24h**
m/z	292.93; 370.05; 360.92; 338.99; 314.66; 340.93; 583.87; 544.90; 598.44; 556.87; 690.83; 758.8; 764.63; 786.63; 870.79; 900.81; 916.79; 278.80; 616.27; 496.34	294.03; 304.93; 408.95; 476.79; 463.85; 544.87; 922.78 968.78; 906.81; 900.08
	**S72h**	**C72h**
m/z	249.16; 214.99; 265.11; 412.93; 476.33; 470.89; 548.84; 532.87; 616.82; 626.85; 600.85; 594.16; 604.86; 610.87; 804.65; 225.47; 356.10;	248.92; 258.90; 232.92;240.98; 264.93; 300.90; 326.91; 278.91; 332.85; 384.86; 368.87; 394.87; 400.84; 452.85; 436.86; 462.86; 468.83; 458.82; 420.85; 478.84; 520.86; 536.82; 542.79; 504.85; 562.80; 558.78; 552.79; 546.82; 510.82; 610.78, 594.80; 598.83; 626.77; 588.82; 678.77; 694.75; 662.85; 642.75; 682.80; 656.79, 672.79; 762.75; 746.77; 730.77; 778.73; 798.76; 750.77; 830.73; 804.79; 814.75; 846.71; 370.93; 354.89; 474.85; 452.86; 304.30;
