# Supplementary figures and images for: Infection with carcinogenic helminth parasites and its production of metabolites induces the formation of DNA-adducts
Source: Infect Agent Cancer. 2019 Nov 29;14:41. doi: 10.1186/s13027-019-0257-2 (PMC6884881; doi:10.1186/s13027-019-0257-2)

# Sample 24h

m/z

**226.95** (242.93; 230.96; 220.94)

**288.82** (294.94; 298.94; 310.91)

362.93 (356.91; 378.90; **361.13**; 377.10; **372.98**; 340.93)

430.91 (424.90; 446.89; **440.87**; **498.90**; 492.89; 514.88; 508.86; 486.87; 530.85)

566.89 (**560.87**; 583.87; 554.86; **576.85**; 538.88; 544.90; 598.44; 556.87; **606.87**)

**634.88** (628.86; 650.85; **644.83**; 666.82; 690.83; **622.84**)

**696.85** (702.87; **718.84**; 712.84; 734.81; 758.8)

764.63 (**786.83**; **770.86**; **780.91**; 758.82)

786.63 (780.81; **802.80**; **838.84**; **848.80**)

**832.82** (854.81; 870.79; 900.81; 916.79)

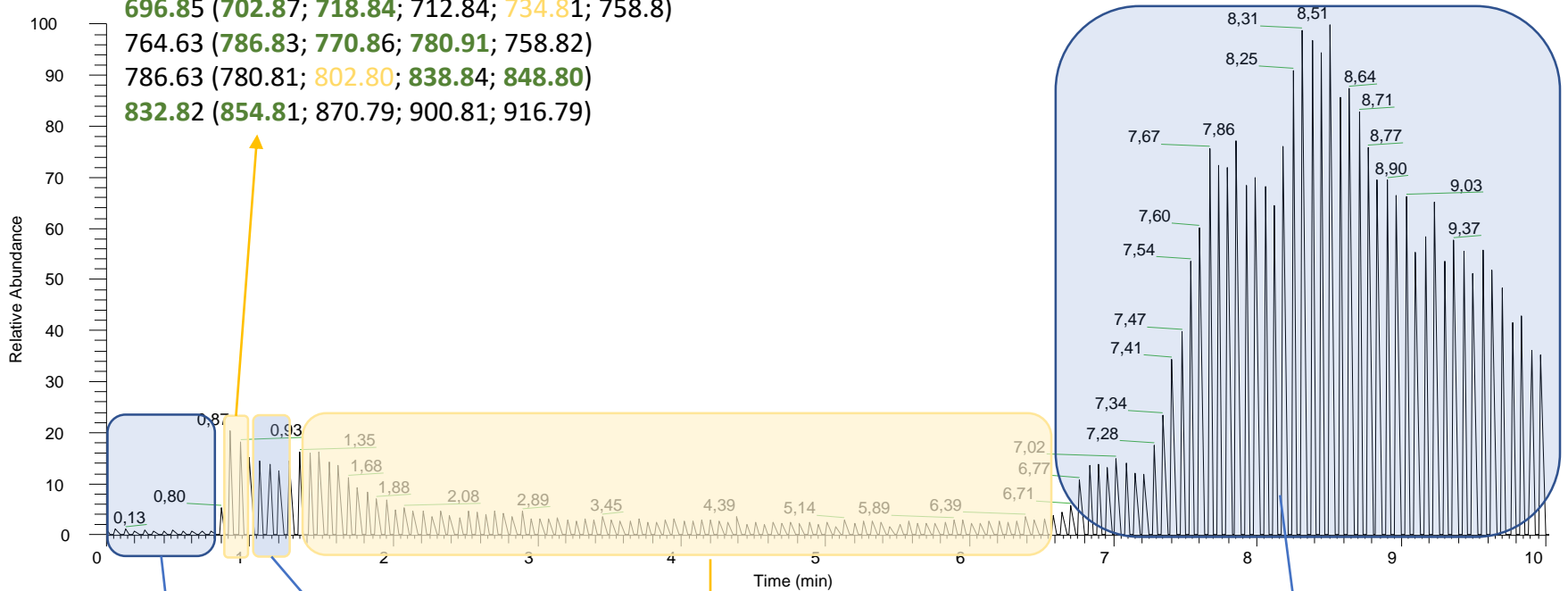

m/z

**240.99**; **225.01**; 319.00; 292.93;

370.05; **230.96**; 360.92; 338.99; 314.66

m/z

**318.94**; **390.94**

m/z

278.80; **256.90**

enzymes

m/z **466.32**; 616.27; 496.34; **404.21**

# control 24h

m/z

**226.96** (242.92; 230.97; **220.94**)

**288.92** (294.03; 310.93; 356.92; 298.95; 304.93; 272.99)

362.98 (378.92; **377.09**; 372.90)

424.96 (430.91; 446.89; **440.88**; 418.95; 408.95)

**498.89** (492.88; 514.87; 508.86; 486.87; 475.20; 476.79; 463.85)

566.88 (560.87; **582.86**; 576.86; 554.86; 544.87)

**628.86** (634.87; 644.83; 650.85; 666.82)

**696.85** (702.85; 718.83; 764.79; **770.83**; **786.81**; **780.91**)

**832.81** (838.82; 854.80; 848.79)

922.78 (968.78; 906.81; 900.08)

RT: 0,00 - 10,01

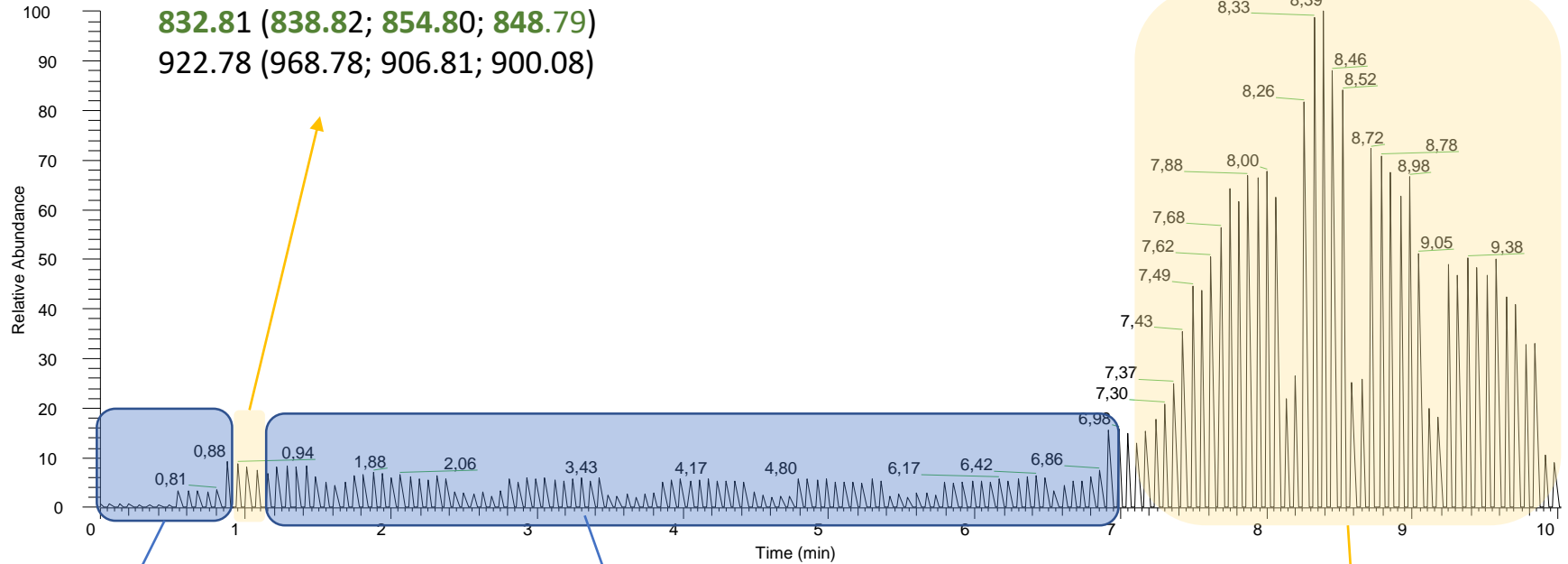

m/z

**204.00; 225.01; 240.99;**

m/z

**390.94; 606.85; 319.00;**

Enzymes

**466.32**

# Sample 72h

m/z

230.96 (226.96; 220.93; 249.16; 214.99)

298.95 (288.92; 272.94; 265.11)

361.13 (377.10; 356.91; 402.90)

418.88 (424.90; 412.93)

475.32 (486.87; 476.33; 470.89; 492.88)

548.84 (542.89; 554.86; 532.87)

616.82 (622.84; 626.85; 600.85; 594.16; 604.86; 610.87; 804.65)

RT: 0.00 - 9.98

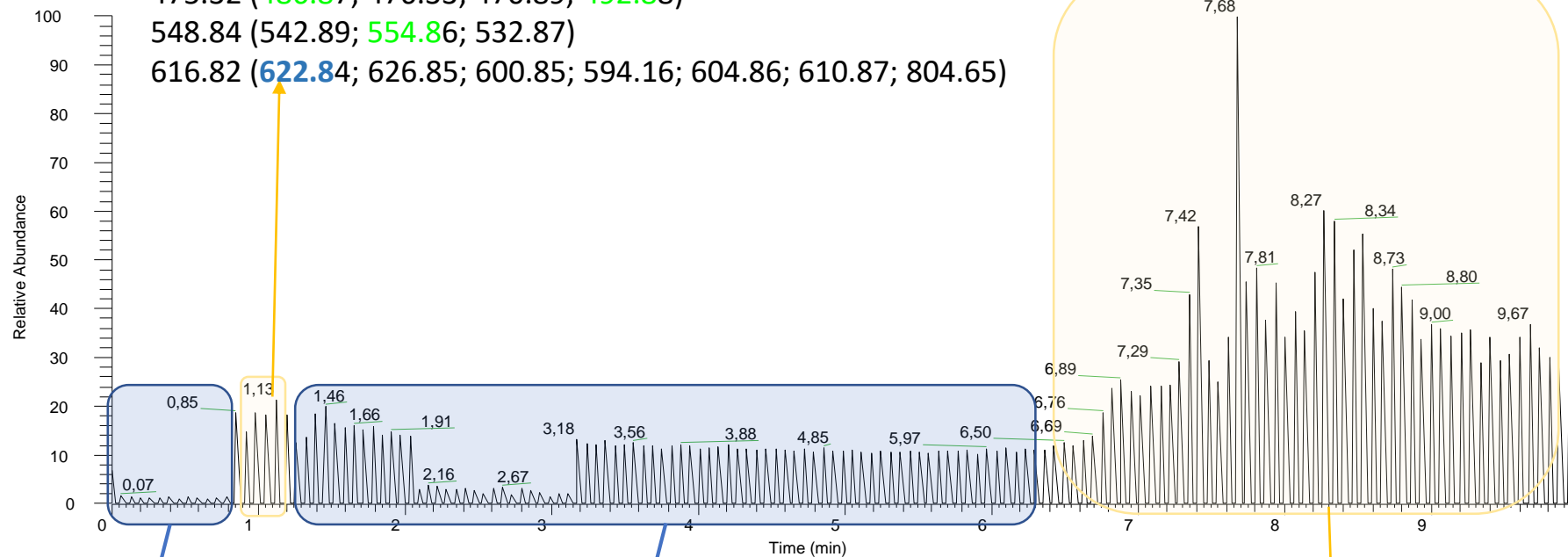

m/z

204.00; 225.47; 319.00

319 (361.13; 377.10; 356.10; 418.88)

enzymes

m/z 466.32 e 404.21

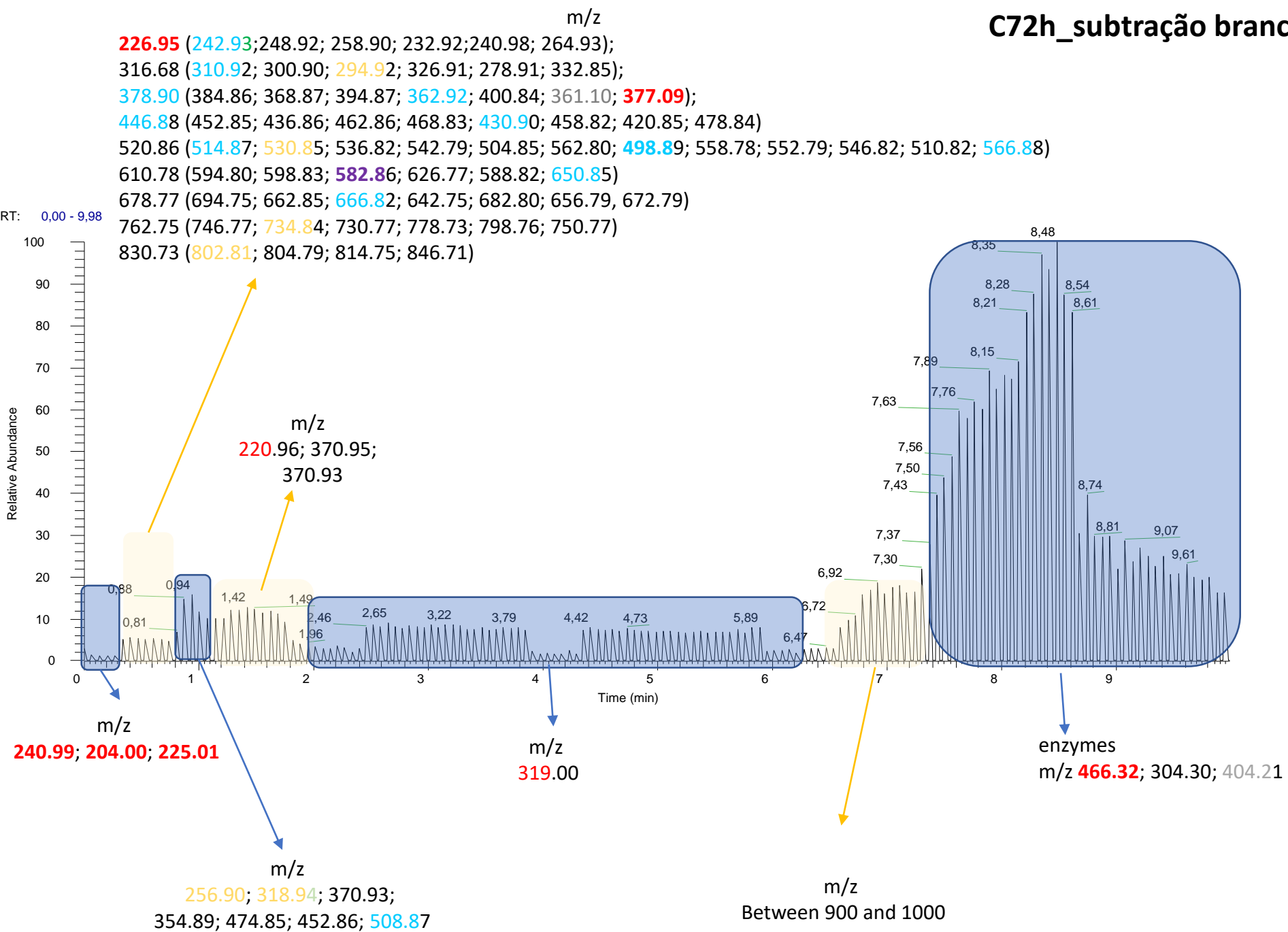

Supplement: Supplementary file 2 — Additional file 2. All m/z detected during analysis of LC-MS/MS of aliquots of samples and control during the course of reaction. Common and exclusive m/z detected for each of aliquots. [file 13027_2019_257_MOESM2_ESM.pdf]
