# Supplementary material for: Infection with carcinogenic helminth parasites and its production of metabolites induces the formation of DNA-adducts
Source: Infect Agent Cancer. 2019 Nov 29;14:41. doi: 10.1186/s13027-019-0257-2 (PMC6884881; doi:10.1186/s13027-019-0257-2)
Supplement: Supplementary file 3 — Additional file 3. Postulated structures for common compounds to different aliquots. [file 13027_2019_257_MOESM3_ESM.pdf]

Common	Postulated structures
**All aliquots**	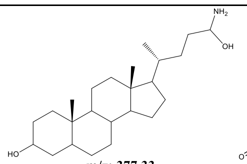 m/z: 377.33 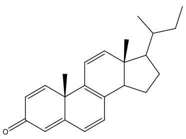 m/z: 320.21 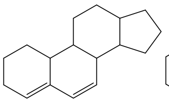 m/z: 228.19 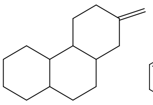 m/z: 204.19 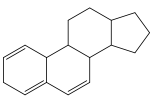 m/z: 226.17 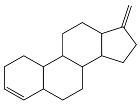 m/z: 242.20 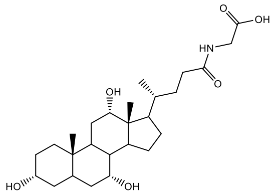 m/z: 465.31)
**S24h and A72h**	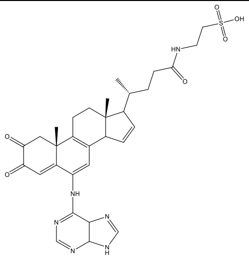 m/z: 622.26
**S24h and C24h**	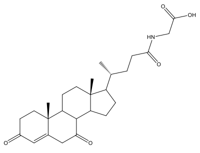 m/z: 439.25 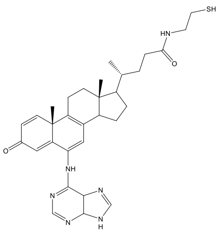 m/z: 560.31 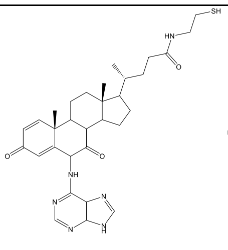 m/z: 578.30 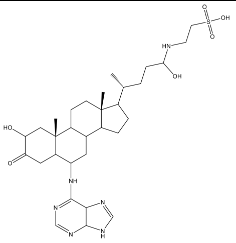 m/z: 634.35 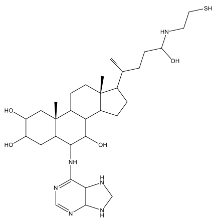 m/z: 606.39 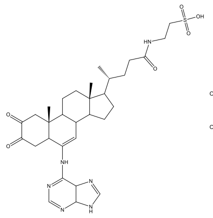 m/z: 628.30 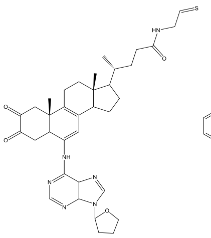 m/z: 646.33 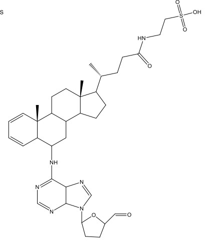 m/z: 696.37 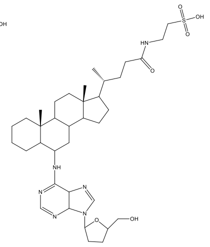 m/z: 702.41 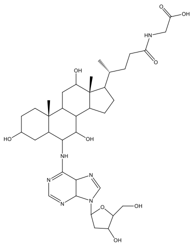 m/z: 718.41 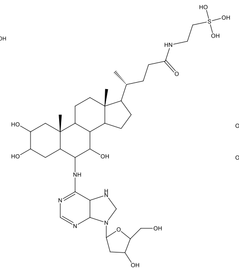 m/z: 770.42 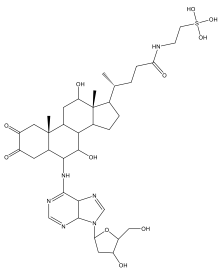 m/z: 780.37 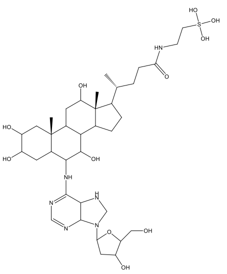 m/z: 786.42 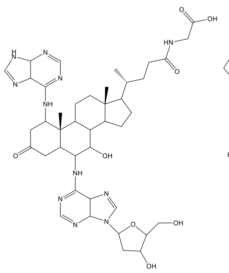 m/z: 833.45 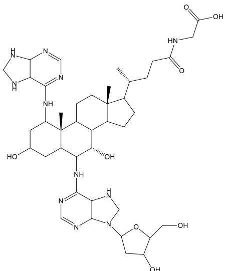 m/z: 839.50 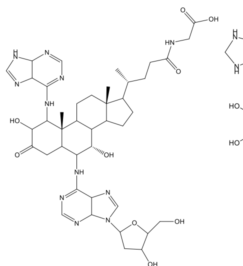 m/z: 849.45 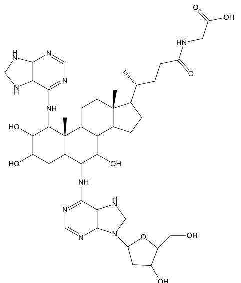 m/z: 855.50

**C72h and S24h**	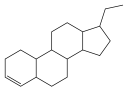 m/z: 258.23 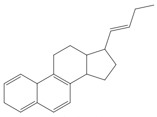 m/z: 278.20 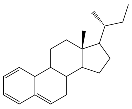 m/z: 296.25 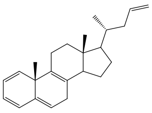 m/z: 320.25 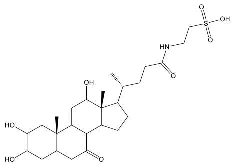 m/z: 531.23 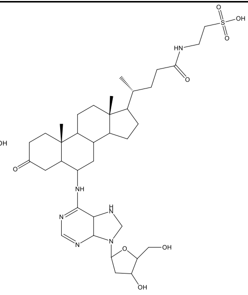 m/z: 734.40 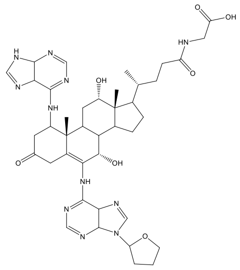 m/z: 801.43
**C24h and S72h**	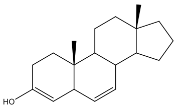 m/z: 272.21 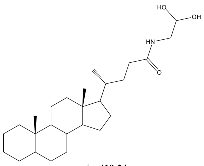 m/z: 419.34 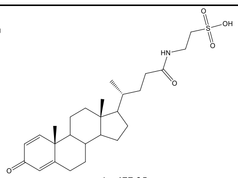 m/z: 477.25 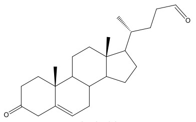 m/z: 356.27
**C72h and C24h**	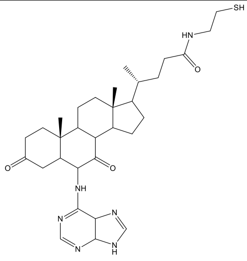 m/z: 582.34
**S72h and C72h**	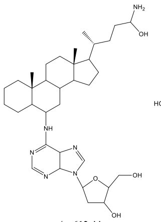 m/z: 612.44 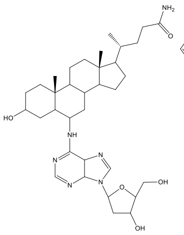 m/z: 626.42 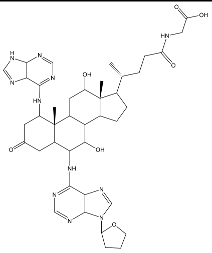 m/z: 803.44
**S24h, S72h, C24h**	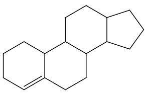 m/z: 230.20 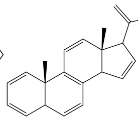 m/z: 288.20 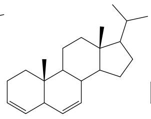 m/z: 298.27 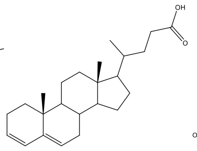 m/z: 356.27 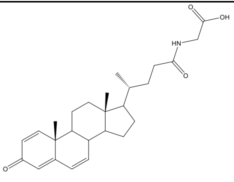 m/z: 425.26 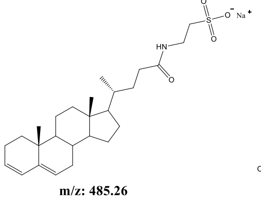 m/z: 485.26 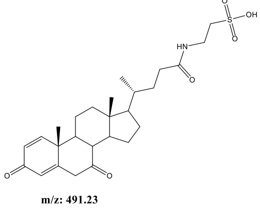 m/z: 491.23 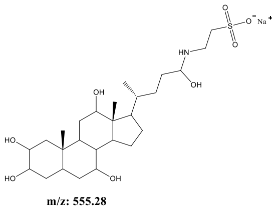 m/z: 555.28

**S72h, S24h, C72h**	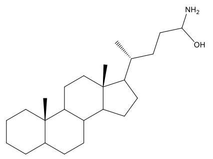 m/z: 361.33 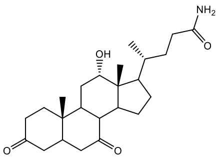 m/z: 403.27
**S24h, C24h, C72h (cont.)**	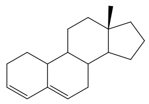 m/z: 242.19 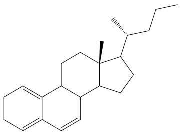 m/z: 310.27 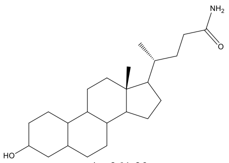 m/z: 361.30 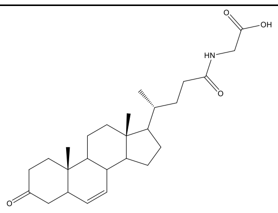 m/z: 429.29 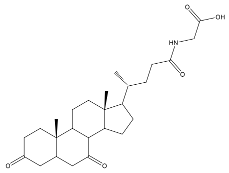 m/z: 445.28 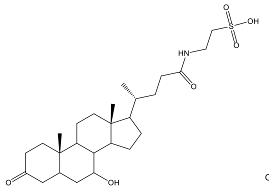 m/z: 497.28 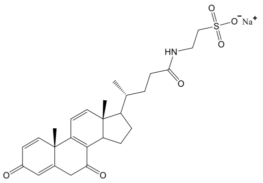 m/z: 509.18 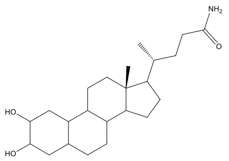 m/z: 377.29 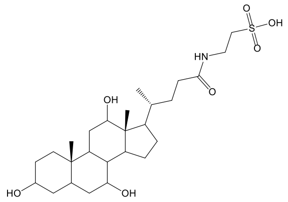 m/z: 515.29 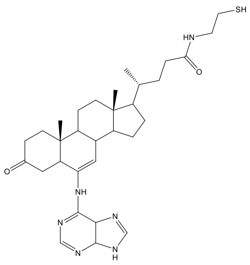 m/z: 566.34 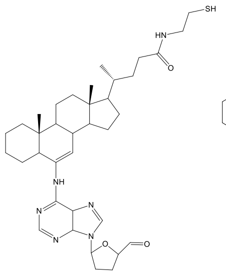 m/z: 650.40 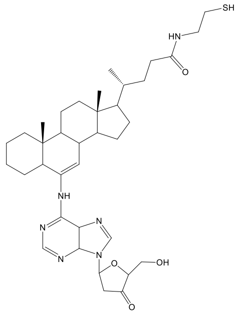 m/z: 666.39
**C24h, C72h, S72h**	Not found.
