# Supplementary material for: Infection with carcinogenic helminth parasites and its production of metabolites induces the formation of DNA-adducts
Source: Infect Agent Cancer. 2019 Nov 29;14:41. doi: 10.1186/s13027-019-0257-2 (PMC6884881; doi:10.1186/s13027-019-0257-2)
Supplement: Supplementary file 4 — Additional file 4. Postulated structures for exclusive m/z detected on samples and control during course of reaction. [file 13027_2019_257_MOESM4_ESM.pdf]

Exclusive	Postulated structures
**S24h (+ CYP)**	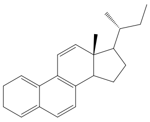 m/z: 292.22 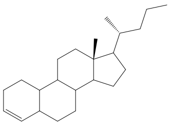 m/z: 314.30 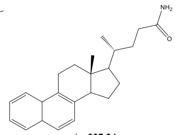 m/z: 337.24 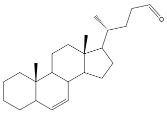 m/z: 342.29 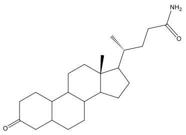 m/z: 359.28 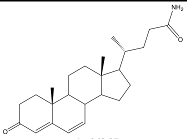 m/z: 369.27 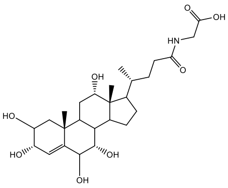 m/z: 495.28 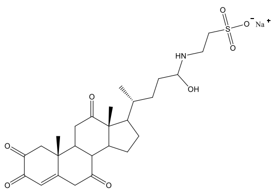 m/z: 545.21 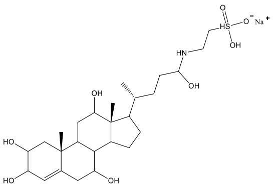 m/z: 555.30 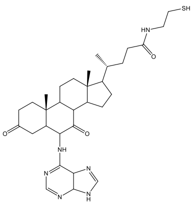 m/z: 582.34 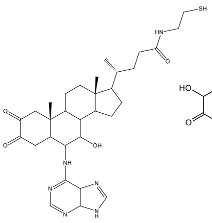 m/z: 598.33 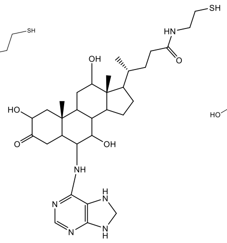 m/z: 616.34 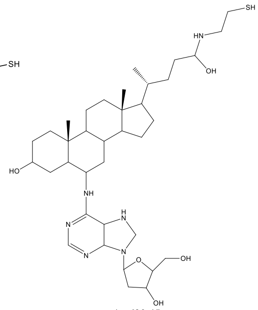 m/z: 690.45 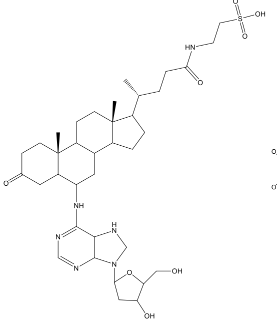 m/z: 734.40 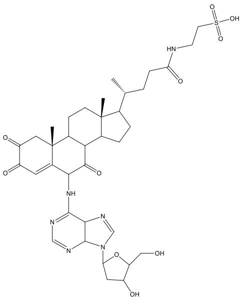 m/z: 758.33 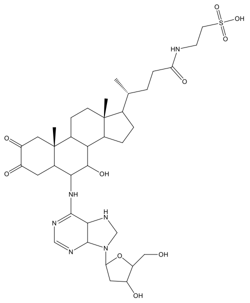 m/z: 764.38 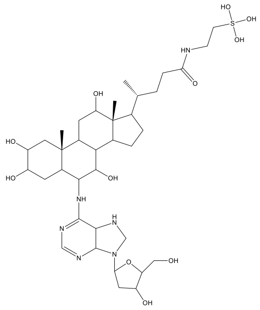 m/z: 786.42 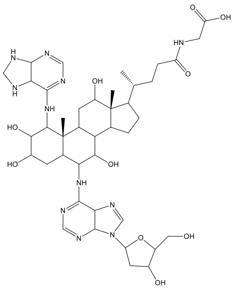 m/z: 869.48 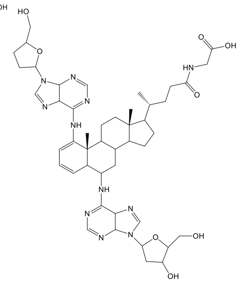 m/z: 899.52 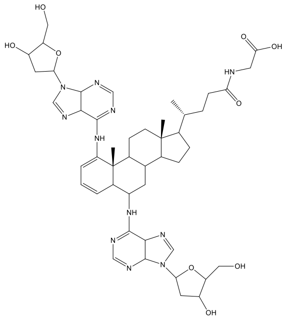 m/z: 915.50
**C24h (-CYP)**	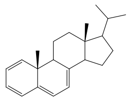 m/z: 294.23 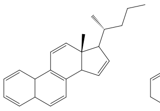 m/z: 304.22 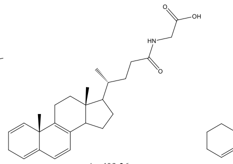 m/z: 409.26 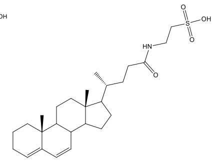 m/z: 463.28 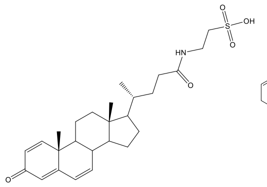 m/z: 475.24 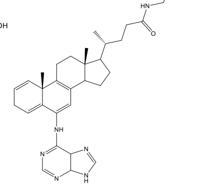 m/z: 544.30 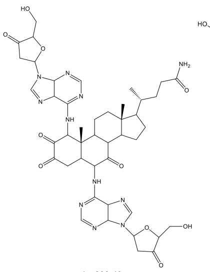 m/z: 899.43 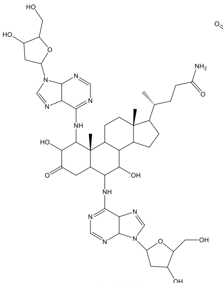 m/z: 907.49 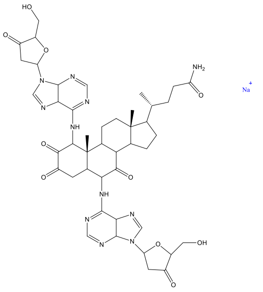 m/z: 922.42 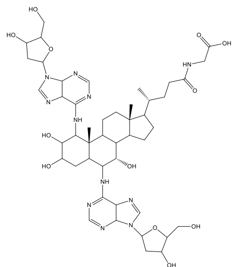 m/z: 967.51

Exclusive	Postulated structures
S72h (+ CYP)	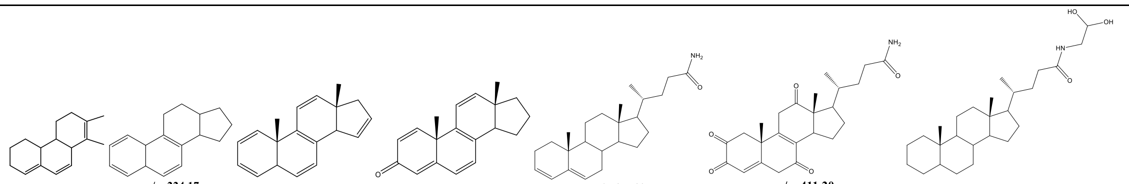<br>m/z: 214.17 m/z: 224.17 m/z: 248.17 m/z: 264.17 m/z: 355.30 m/z: 411.20 m/z: 419.34<br>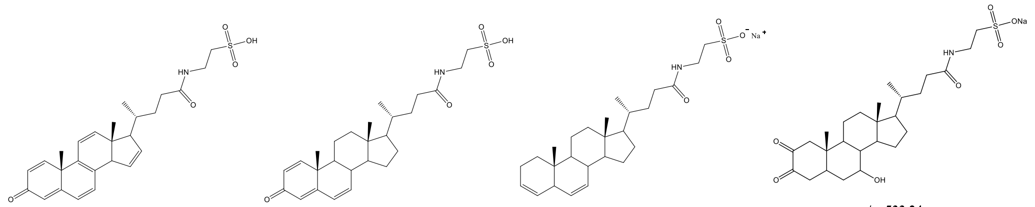<br>m/z: 469.19 m/z: 475.24 m/z: 485.26 m/z: 533.24<br>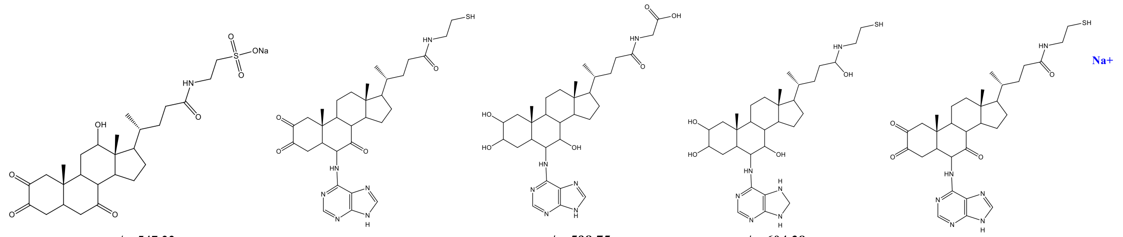<br>m/z: 547.22 m/z: 594.30 m/z: 598.75 m/z: 604.38 m/z: 617.29
C72h (- CYP)	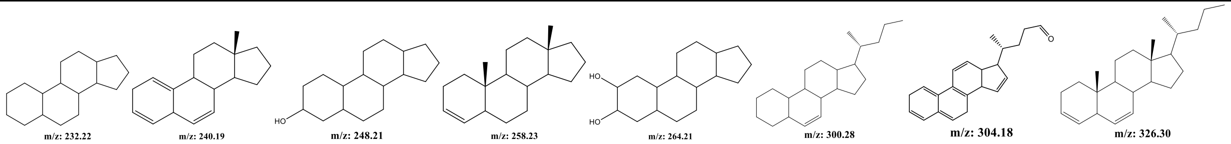<br>m/z: 232.22 m/z: 240.19 m/z: 248.21 m/z: 258.23 m/z: 264.21 m/z: 300.28 m/z: 304.18 m/z: 326.30<br>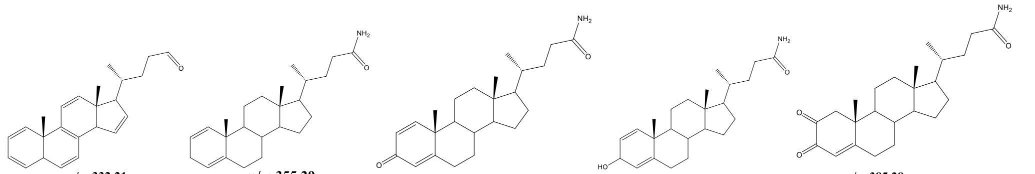<br>m/z: 332.21 m/z: 355.29 m/z: 369.25 m/z: 371.25 m/z: 385.28<br>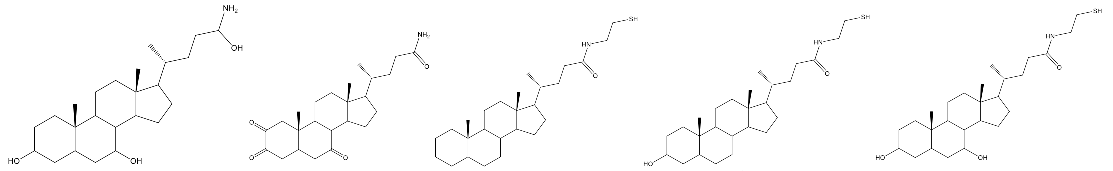<br>m/z: 393.32 m/z: 401.26 m/z: 419.32 m/z: 435.32 m/z: 451.31<br>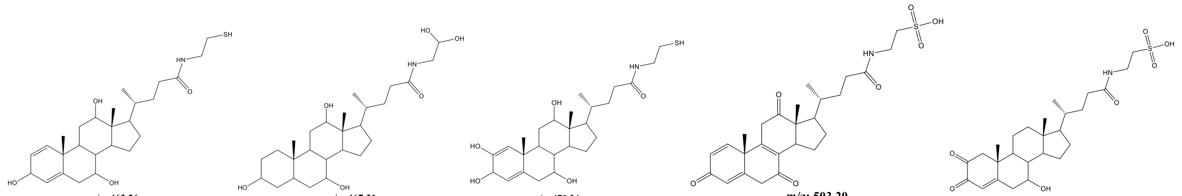<br>m/z: 463.26 m/z: 467.31 m/z: 479.24 m/z: 503.20 m/z: 509.24<br>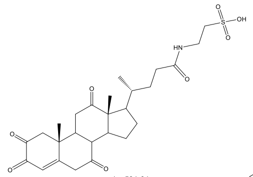<br>m/z: 521.21<br>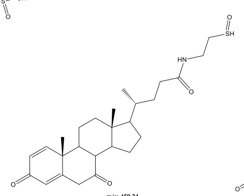<br>m/z: 459.24<br>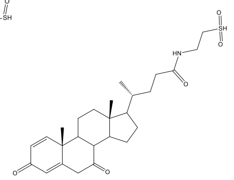<br>m/z: 475.24

Exclusive	Postulated structures
**C72h (- CYP)**	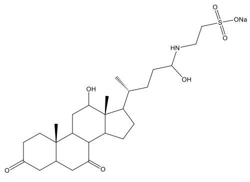<br>m/z: 535.27<br>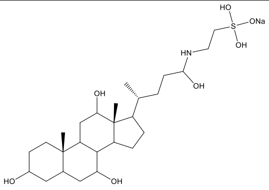<br>m/z: 541.30<br>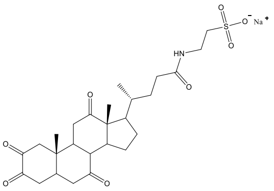<br>m/z: 545.21<br>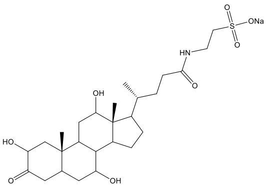<br>m/z: 551.25<br>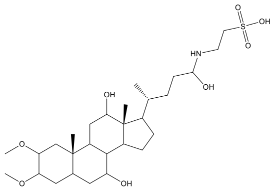<br>m/z: 557.30<br>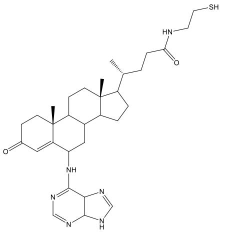<br>m/z: 566.36<br>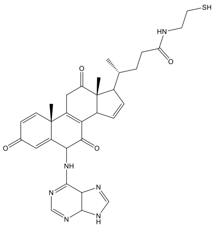<br>m/z: 588.25<br>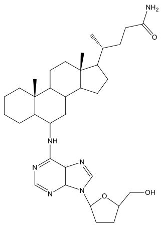<br>m/z: 594.43<br>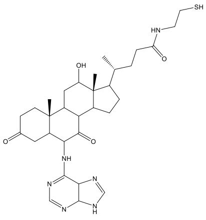<br>m/z: 598.33<br>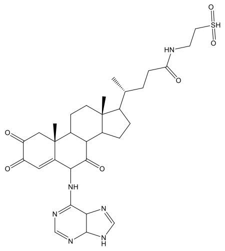<br>m/z: 626.29<br>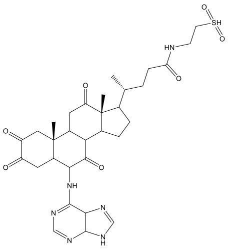<br>m/z: 642.30<br>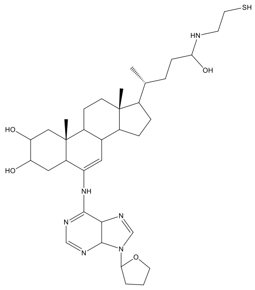<br>m/z: 656.41<br>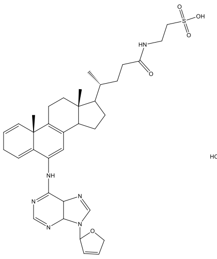<br>m/z: 662.33<br>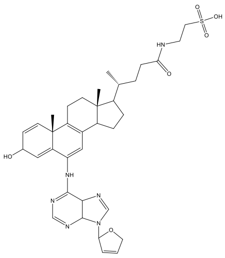<br>m/z: 678.32<br>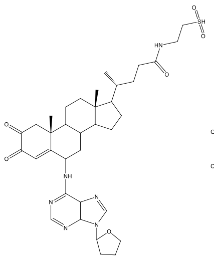<br>m/z: 682.34<br>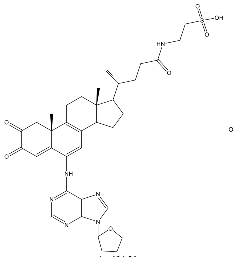<br>m/z: 694.31<br>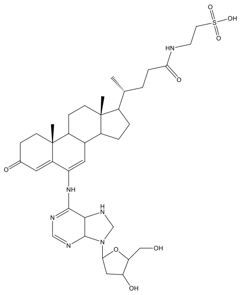<br>m/z: 730.37<br>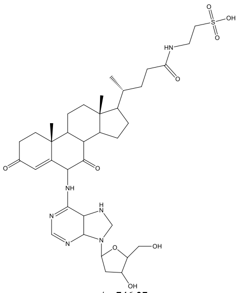<br>m/z: 746.37<br>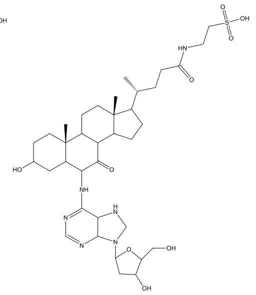<br>m/z: 750.40<br>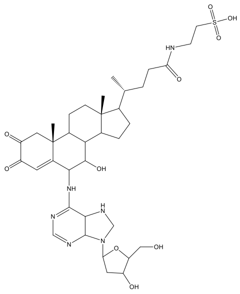<br>m/z: 762.38<br>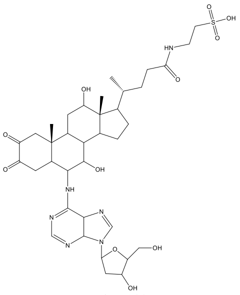<br>m/z: 778.36<br>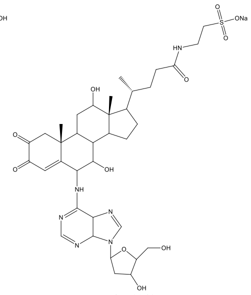<br>m/z: 798.32<br>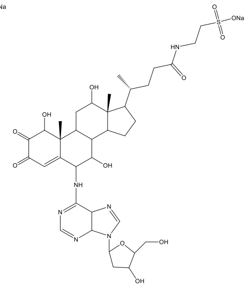<br>m/z: 814.33<br>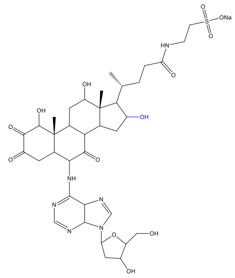<br>m/z: 830.31<br>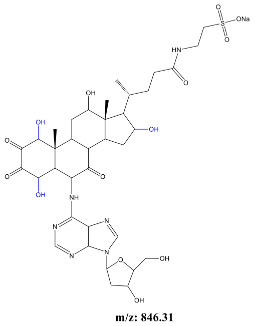<br>m/z: 846.31
